# Subregional CT radiomics for preoperative prediction of mitotic index and risk stratification in 2-5 cm gastrointestinal stromal tumors of the stomach: a dual-center study

**DOI:** 10.3389/fonc.2025.1708058

**Published:** 2026-01-15

**Authors:** Gang Peng, Peiyun Zhu, Buhan Zhang, Zening Zhang, Youjun Mao, Risheng Yu, Jihong Sun

**Affiliations:** 1 Department of Radiology, Guangde Traditional Chinese Medicine Hospital, Guangde, Anhui, China; 2 Department of Radiology, The Second Affiliated Hospital of Jiaxing University, Jiaxing, Zhejiang, China; 3 School of Medical Imaging, Bengbu Medical University, Bengbu, Anhui, China; 4 Department of Radiology, Sir Run Run Shaw Hospital, Zhejiang University School of Medicine, Hangzhou, Zhejiang, China; 5 Hangzhou Purui Medical Technology Co., Ltd, Hangzhou, Zhejiang, China; 6 Department of Radiology, The Second Affiliated Hospital, Zhejiang University, School of Medicine, Hangzhou, Zhejiang, China

**Keywords:** computed tomography, gastrointestinal stromal tumors, mitotic index, radiomics, subregional

## Abstract

**Purpose:**

To propose a model based on computed tomography (CT) subregional radiomics to predict the preoperative mitotic index of 2-5 cm Gastrointestinal Stromal Tumors (GISTs) of the stomach.

**Materials and methods:**

This retrospective study enrolled a total of 368 patients with GISTs from two institutions: Center 1 comprised 239 patients (122 M, 117 F; mean age 61.66 ± 10.86 years), and Center 2 comprised 129 patients (51 M, 78 F; mean age 60.28 ± 9.72 years). Radiomics features were extracted from the entire tumor. Concurrently, k-means clustering was applied to imaging features to define three distinct tumor subregions, from which radiomics features were subsequently extracted. The Recursive Feature Addition method was used to identify features correlated with the mitotic index in patients with 2-5 cm gastric GISTs. Using the selected features from each subregion and the whole tumor, logistic regression (LR) was employed to construct subregion-based radiomics models and conventional whole-tumor-based radiomics models, respectively.

**Results:**

Better performance was observed for unenhanced CT subregions 1, 2, and 3 compared with the conventional radiomics model. The area under the receiver operating characteristic curve (AUC), accuracy, sensitivity, and specificity of the model for subregion 3 in the training set were 0.98, 0.97, 0.98, and 0.90, respectively. In the validation and external test sets, the AUC values were 0.874 and 0.804, respectively. The conventional whole-tumor radiomics model based on venous phase CT demonstrated superior performance compared to all subregion-based models, achieving an AUC of 0.956 in the training set, with accuracy, sensitivity, and specificity of 0.94, 0.97, and 0.83, respectively. In the validation and external test sets, it attained AUC values of 0.892 and 0.805, respectively.

**Conclusion:**

Subregional CT radiomics may be used to predict the mitotic index of patients with 2-5 cm gastric GIST before surgery. In particular, subregional radiomics models based on unenhanced CT showed excellent predictive performance.

## Introduction

Gastrointestinal stromal tumors (GISTs) are the most common mesenchymal tumors in the gastrointestinal tract, with an incidence of 1/100,000 (1, 2). GISTs can emerge in any part of the gastrointestinal tract, with a higher prevalence in the stomach (60–65%) (1). Currently, endoscopic resection of 2-5 cm gastric GISTs has been clinically implemented due to its advantages such as minimal invasiveness and rapid recovery, offering potential benefits to patients (3–5). However, this therapeutic approach carries the risk of incomplete tumor resection, resulting in a higher positive margin rate and consequently increasing the likelihood of postoperative recurrence in patients (3). Meanwhile, some studies have suggested that laparoscopic or open surgical resection of 2-5 cm gastric GISTs is a suitable treatment approach (6–8). Therefore, clinicians face challenges in choosing treatment strategies for 2-5 cm gastric GISTs. Risk stratification of 2-5 cm gastric GISTs is primarily determined by the mitotic index (9). Studies have demonstrated that in 2-5 cm gastric GISTs, the metastasis rates are 16% and 1.9% for patients with mitotic indices >5/50 high-power fields (HPF) and ≤5/50 HPF, respectively (10). The mitotic index is typically determined from postoperative pathology. Although it can be obtained preoperatively through biopsy, the limited sample size often fails to represent the entire tumor profile, frequently leading to an underestimation of the recurrence risk (11). Therefore, exploring a noninvasive and reliable method for the preoperative prediction of malignant potential in patients with gastric GIST holds significant clinical value.

Computed tomography (CT) is a widely used imaging method for the diagnosis, efficacy evaluation, and postoperative follow-up of GISTs owing to its advantages of noninvasiveness and convenience (12). Studies have shown that analyzing clinical and CT imaging features can provide certain diagnostic value for preoperatively assessing the risk of 2-5 cm GISTs (13). However, the assessment of imaging features is largely subjective, and results in variability among evaluators. Compared to traditional imaging features, radiomics can extract high-dimensional quantitative features that are difficult to discern with the naked eye, thereby providing support for clinical decision-making (14–16). Current research indicates that conventional radiomics based on whole-tumor CT imaging has shown some efficacy in predicting the mitotic index and risk stratification of GISTs (17–20). Conventional radiomics mainly focuses on the overall characteristics of tumors. In recent years, studies have utilized Gaussian mixture models to cluster intratumoral features, delineating subregions with distinct biological characteristics. Predictive models constructed based on these subregional features have demonstrated excellent performance (21, 22). This method quantifies tumor heterogeneity, thereby addressing the shortcomings of conventional radiomic studies that neglect important information (23).

Therefore, the aim of this study is to explore the construction of predictive models based on CT subregional radiomics to predict the preoperative mitotic index of 2-5 cm gastric GISTs. The goal was to achieve preoperative risk stratification of GISTs and implement individualized precision medicine.

## Materials and methods

### Study groups

This retrospective study was approved by the Ethics Committee of Sir Run Run Shaw Hospital, Zhejiang University School of Medicine. This study was exempt from the requirement for informed consent from patients. The study continuously collected clinical and imaging data from patients with gastric GISTs, confirmed by postoperative pathological diagnosis, from January 2016 to December 2023 at Sir Run Run Shaw Hospital (Center 1) and the Second Affiliated Hospital, Zhejiang University School of Medicine (Center 2).

The criteria for patient involvement were as follows: (1) endoscopic or surgical resection; (2) histopathologically confirmed gastric GISTs; and (3) unenhanced and enhanced CT scans performed within 2 weeks prior to surgery. The exclusion criteria were as follows: (1) treatment with targeted drugs such as imatinib before surgery; (2) preoperative metastatic or recurrent GISTs; (3) presence of other malignancies or detection of multiple GISTs; (4) incomplete clinical and imaging data or poor-quality CT images; and (5) tumor diameter <2 cm or >5 cm.

**Figure 1:**
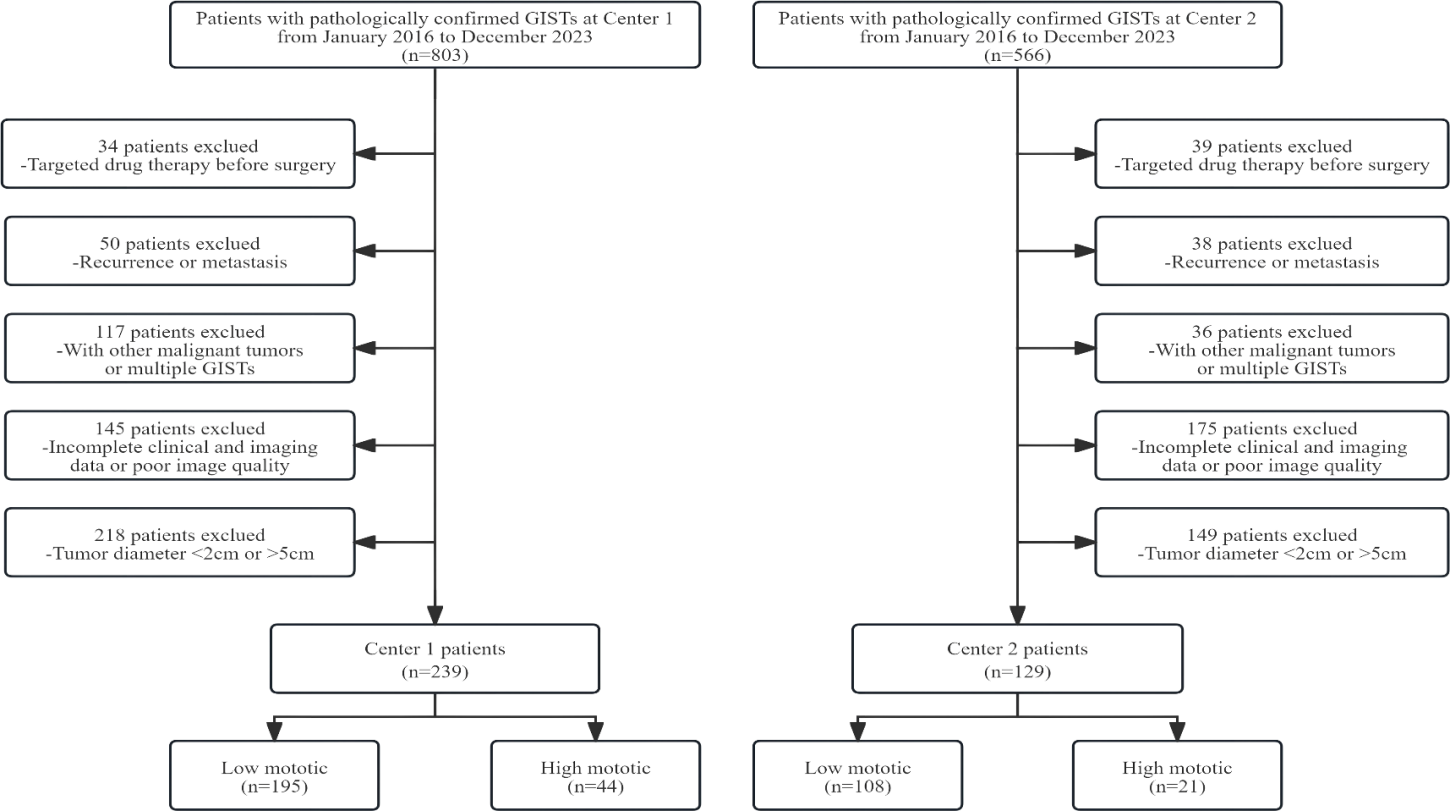
The flowchart of patient enrollment. GISTs, gastrointestinal stromal tumors.

Ultimately, this study included 239 patients with 2-5 cm GISTs from Center 1 as the training/validation group and 129 patients from Center 2 as the external testing group. Based on the mitotic index values from the pathological results, patients were grouped into low mitotic group with mitotic index ≤ 5 and high mitotic group with mitotic index > 5. The screening process for the study participants is shown in **Figure 1**.

### Clinical data and pathological criteria

By retrieving data from the electronic medical record systems of the two hospitals, we collected information on patient sex, age, body mass index (BMI), and preoperative laboratory tumor markers. The tumor marker data included alpha-fetoprotein (AFP), carcinoembryonic antigen (CEA), carbohydrate antigen 19-9 (CA 19-9), and carbohydrate antigen 125 (CA 125). The pathological diagnosis for risk stratification was based on the NIH 2008 Consensus Classification system (9).

### CT image acquisition

The imaging equipment used in this study included the SOMATOM Definition Edge, SOMATOM Definition AS, SOMATOM Definition Flash, and Sensation 16 (Siemens, Germany) as well as the LightSpeed VCT, Revolution Maxima, and Optima CT620 (GE, USA). The patients were required to fast for 6–8 h before the examination and were instructed to drink 600–1000 mL of warm water 15 min before the scan to adequately distend the gastric cavity. The scanning range focused on the upper mid-abdomen and encompassed all lesion areas. Scanning parameters such as a tube voltage of 120 kV, automatic tube current, axial image slice thickness of 5 mm, and multiplanar reconstruction slice thickness of 1.5–2 mm, were configured. After completing the unenhanced scan, a non-ionic iodinated contrast agent was injected using a high-pressure injector for the enhanced scan, at a dose of 1.5–2.0 mL/kg and an injection rate of 2.5–3.5 mL/s. Arterial-phase scans were performed 25–30 seconds after contrast injection, followed by venous-phase scans 50–60 seconds later.

### CT image evaluation and segmentation

The CT images of the patients were generated using the Picture Archiving and Communication System (PACS) in the DICOM format. Two abdominal radiologists analyzed the location and size of the tumors on the CT images. They had 10 and 15 years of experience in abdominal imaging, respectively, and were blinded to the clinical and pathological information. The tumor locations were categorized as the cardia, fundus, body, and antrum of the stomach. The maximum diameter of the multiplanar reconstructed images represents the tumor size.

All exported images were anonymized. Image segmentation was performed by two radiology residents with 5 years of experience in abdominal diagnostics using the ITK-SNAP software (version 3.8, https://www.itksnap.org). The residents manually outlined the tumors on the unenhanced and venous-phase CT images layer-by-layer with the aim of including all tumor areas. Adjacent normal gastric mucosal tissue and nearby blood vessels were avoided to obtain the tumor’s region of interest (ROI). Subsequently, another radiologist with more than 10 years of experience in abdominal diagnostics reviewed and corrected the outlines. In the case of discrepancies, discussions were held with a senior radiologist with 15 years of experience to reach a consensus. One month later, 30 samples were randomly selected for a second set of measurements and ROI outlines. Inter- and intra-observer consistency in tumor measurements and ROI outlines were assessed using the intraclass correlation coefficient (ICC > 0.80) and the Dice similarity coefficient (Dice > 0.80), respectively.

### Radiomics feature extraction

Variations in scanning machines, reconstruction slice thicknesses, and pixel sizes can cause differences in imaging characteristics. Accordingly, all CT images were subjected to Z-score normalization preprocessing before feature extraction to minimize the differences. The mean intensity and standard deviation of CT images were calculated. Each image was normalized using a standardized formula to achieve a mean intensity of approximately 0 and a standard deviation of 1. No additional harmonization methods, such as ComBat or other batch-effect correction techniques at the feature level, were applied. The voxel size was standardized to 1 mm × 1 mm × 1 mm³. The K-means algorithm was used to cluster the ROI image features. The optimal number of clusters, N, was determined by iteratively computing the average AUC values across different values of N. To ensure reproducibility, the following initialization strategy was applied to the normalized input images prior to K-means subregion clustering: the normalized intensity values [0, 1] were divided into N (N = 3–5) equal-width bins; the mean intensity of voxels within each bin was computed and used as the initial centroids. Subsequently, K-means clustering was performed based on these initial centroids to obtain N tumor subregions. The optimal number of clusters was determined to be three (K = 3), resulting in three tumor subregions designated as subregions 1, 2, and 3. Subsequently, radiomic features were extracted using PyRadiomics (version 3.1; https://pyradiomics.readthedocs.io). Radiomic features were extracted from the entire tumor and its subregions on both unenhanced and venous phase CT images, including: (1) first-order statistical features, capturing intensity-based histogram metrics (18 features); (2) shape features, describing tumor geometry and morphology (14 features, extracted only from the original images); (3) texture features, quantifying spatial relationships via gray-level co-occurrence matrix (GLCM; 24 features), gray-level dependence matrix (GLDM; 14 features), gray-level run-length matrix (GLRLM; 16 features), and gray-level size zone matrix (GLSZM; 14 features), totaling 68 features per set. (4) additional transformation-based features, which utilized wavelet transforms to extract higher-order features, including the Laplacian of Gaussian (LoG) filter and local binary patterns (LBP) (each with 18 first-order and 68 texture features). In total, this resulted in 1,045 features per lesion across all image sets. All features extracted from the unenhanced and venous phase CT images accorded with the imaging features which was compatible with the International Biomarker Standardization Initiative (IBSI) (24).

### Feature selection and model construction

**Figure 2:**
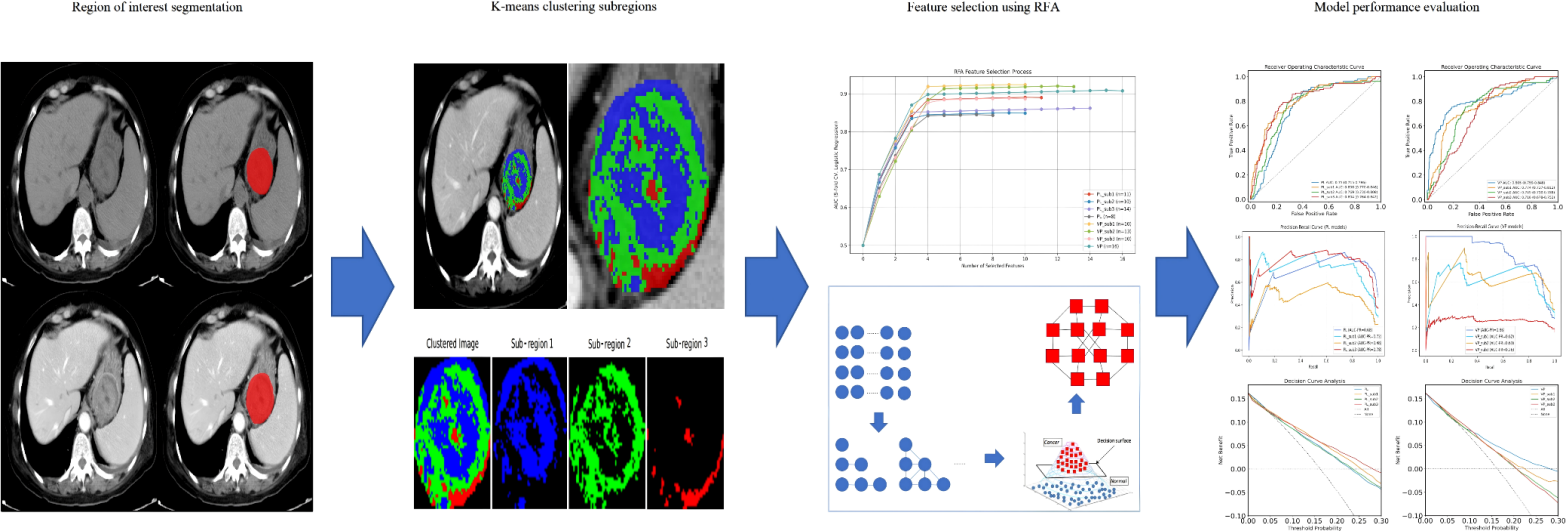
Overview of the workflow in the study. RFA, recursive feature addition.

Spearman’s correlation analysis (ICC > 0.75) was employed to remove highly correlated and redundant radiomic features. Previous studies have utilized Recursive Feature Elimination (RFE) (25) for feature selection, which can identify the most informative features by gradually reducing the number of features. The RFE process involves removing the least important features based on the model’s feature weights or coefficients in each iteration, followed by retraining the model until the desired number of features is achieved or a specific performance metric is satisfied. This approach can be integrated with various machine learning models such as support vector machines, decision trees, and random forests. Conversely, Recursive Feature Addition (RFA) employs the inverse principle of RFE to optimize the feature subset by progressively adding features. This method begins with an empty feature set, attempts to add the remaining unselected features at each step, and evaluates the performance of the model after each addition. The dimensionality of the preprocessed features can be reduced using RFA, and an optimized feature subset is generated to construct the predictive model. Based on the features from subregions 1, 2, 3, and the whole tumor after screening, as well as Logistic Regression (LR), we constructed a total of eight prediction models using unenhanced and venous-phase-enhanced CT data. Given the limited sample size, we did not separate the internal validation set from the training data. Instead, we employed a fivefold cross-validation method, which allowed for better utilization of data to build a stable model and provided a more reliable assessment of model performance (26). To mitigate class imbalance in the dataset, we applied random oversampling to the minority class. This involved randomly selecting instances from the minority class with replacement until the class distribution reached approximate balance, typically at a 1:1 ratio with the majority class. The models ware validated using an external test cohort. We also developed a clinical model for comparison. The process of this study is illustrated in **Figure 2**.

### Model evaluation and interpretability

Receiver Operating Characteristic (ROC) curves were used to evaluate the predictive models, and the area under the curve (AUC) was calculated along with accuracy, sensitivity, and specificity. Additionally, the utility of the predictive models in clinical decision-making was assessed using Decision Curve Analysis (DCA).

Although radiomics models achieve high accuracy, their practical application is hindered by the difficulty of reasonably interpreting the contribution of individual features to the model. The SHAP (Shapley Additive Explanations) method is an additive decomposition technique that visualizes model results by computing feature importance values (SHAP values), thereby explaining each radiomic feature’s contribution to the prediction (27). This is an ideal tool for model interpretation and visualization. Therefore, this study introduced the SHAP method to evaluate the importance of radiomics features within the model and perform visual analysis, thereby enhancing the interpretability of the constructed radiomics model.

### Statistical analysis

SPSS software (version 26.0, IBM, USA) was used for statistical analysis. For continuous variables, differences between the two groups were evaluated using the t-test or Mann-Whitney U test, and the specific method depended on the data distribution. The chi-square test or Fisher’s exact test was used to compare differences among categorical variables. A *p*-value lower than 0.05 illustrated the statistical significance.

## Results

### Clinical characteristics

A total of 239 patients with 2–5 cm GISTs at Center 1 were enrolled in this study, comprising 122 males and 117 females. Among these patients, there were 195 cases of low mitotic and 44 cases of high mitotic, with a mean age of 61.66 ± 10.86 years old (ranging from 29 to 86 years old). Center 2 included 129 patients (51 males and 78 females). Wherein, there were 108 cases of low mitotic and 21 cases of high mitotic, with a mean age of 60.28 ± 9.72 years old (ranging from 34 to 80 years old). In terms of clinical characteristics, significant differences were observed between the two patient groups in terms of sex (*P* = 0.035) and CA199 (*P* < 0.001). No significant differences were observed in the other clinical or CT features (*P* > 0.05). The analysis of the clinical and CT characteristic indicators is summarized in **Table 1**.

**Table 1:** Statistical analysis of clinical and CT characteristics of all patients in Center 1 and Center 2.

	Center 1 n=239			Center 2 n=129			*P*
Item	Mitotic index ≤5 [n=195 (%)]	Mitotic index >5 [n=44 (%)]	*P*	Mitotic index ≤5 [n=108 (%)]	Mitotic index >5 [n=21 (%)]	*P*	
Sex			0.411			0.408	0.035*
Male	102(52.31)	20(45.45)		67(62.04)	11(52.38)		
Female	93(47.69)	24(54.55)		41(37.96)	10(47.62)		
Age (years)	62.39 ± 10.73	58.41 ± 10.96	0.022*	60.97 ± 9.39	56.71 ± 10.82	0.130	0.275
BMI (kg/m2)	23.83 ± 3.35	23.46 ± 2.54	0.764	23.30 ± 3.53	23.30 ± 3.93	0.594	0.157
CEA (ng/mL)	2.49 ± .1.70	2.32 ± 1.41	0.672	2.42 ± 1.62	1.78 ± 0.92	0.085	0.255
AFP (ng/mL)	2.84 ± 1.68	3.75 ± 4.69	0.553	2.95 ± 1.49	2.41 ± 0.81	0.237	0.395
CA19-9 (IU/mL)	12.60 ± 17.94	13.66 ± 13.58	0.520	7.35 ± 6.34	5.16 ± 3.61	0.104	<0.001*
CA125 (U/mL)	10.65 ± 7.82	10.45 ± 3.44	0.089	12.36 ± 8.92	10.33 ± 6.62	0.159	0.072
Location			0.161			0.360	0.386
Cardia	2(1.03)	2(4.55)		4(3.70)	1(4.76)		
Fundus	68(34.87)	20(45.45)		38(35.19)	11(52.38)		
Body	102(52.31)	19(43.18)		59(54.63)	7(33.33)		
Antrum	23(11.79)	3(6.82)		7(6.48)	2(9.53)		
Tumor Size (cm)	3.29 ± 0.86	3.55 ± 0.89	0.088	3.19 ± 0.90	3.65 ± 0.88	0.039*	0.396

BMI, body mass index; CEA, carcinoembryonic antigen; AFP, alpha fetoprotein; CA19-9, carbohydrate antigen 19-9; CA125, carbohydrate antigen 125; *, *P* < 0.05. The values here have been bolded.

### Selected radiomics feature

**Figure 3:**
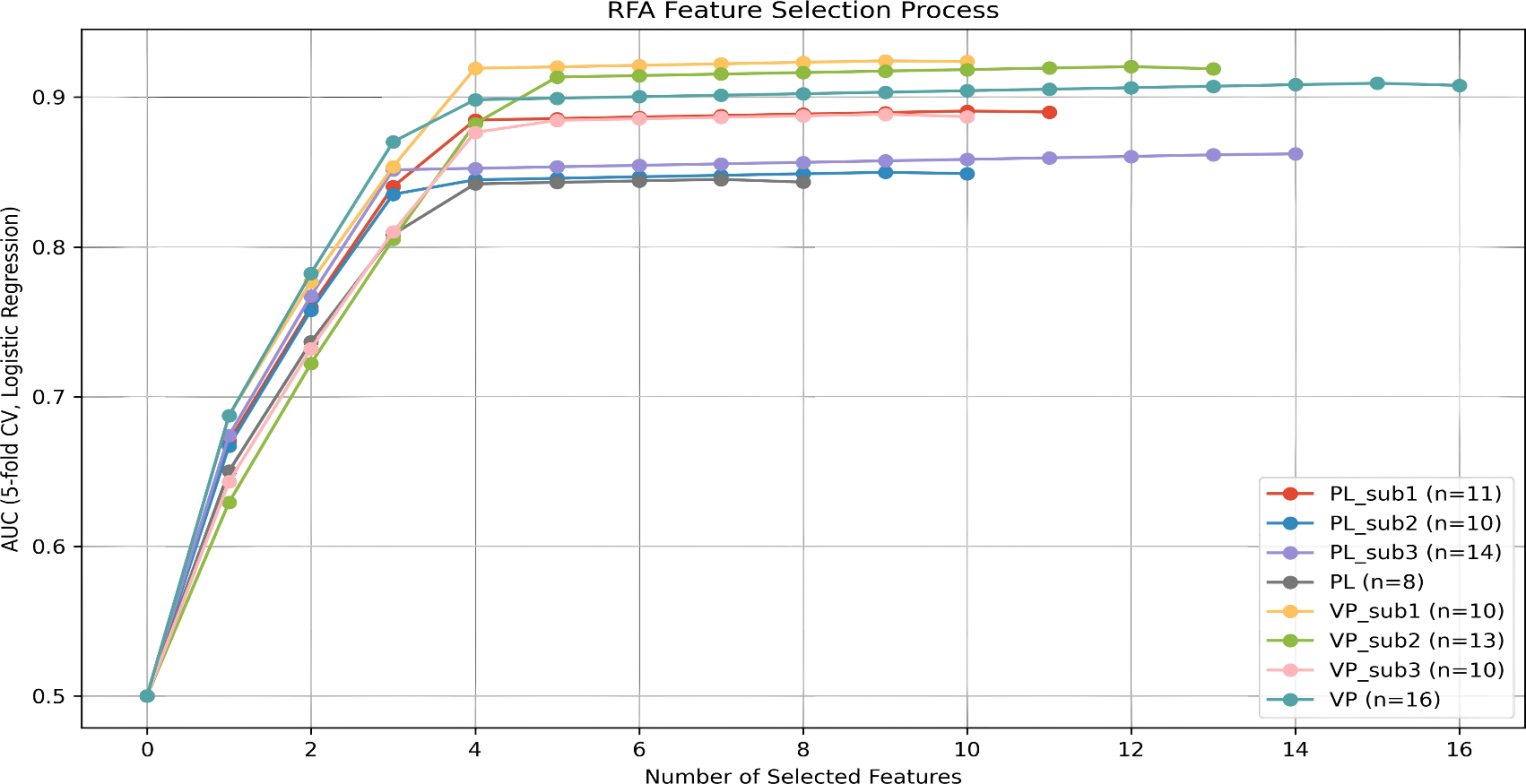
The important radiomics features extracted by RFA from unenhanced and venous phase CT imaging were retained in the following quantities per region:Unenhanced phase: subregion 1 (11); subregion 2 (10); subregion 3 (14); and whole tumor region (8).Venous phase: subregion 1 (10); subregion 2 (13); subregion 3 (10); and whole tumor region (16).RFA, recursive feature addition; PL, plain scan/unenhanced scan; VP, venous phase.

Spearman correlation analysis was employed to eliminate redundant features, with feature pairs exhibiting ICC > 0.75 and an absolute correlation coefficient |ρ| > 0.9 being identified, and one feature from each such pair subsequently removed. Thereafter, RFA was independently applied to each imaging group (subregion 1, subregion 2, subregion 3, and the entire tumor region for both the unenhanced and venous phases, yielding a total of 8 feature sets in all). The specific steps of RFA were as follows: initiation from an empty feature set; in each iteration, sequential evaluation of adding each remaining candidate feature to the current set; utilization of logistic regression as the base classifier, with 5-fold cross-validation performed on the training cohort to compute the AUC following each addition; permanent incorporation of the feature yielding the maximum enhancement in mean AUC; and repetition of the addition process until satisfaction of one of the following termination criteria: further addition of any feature failed to increase the mean AUC from 5-fold cross-validation by more than 0.001 (the predefined tolerance threshold), or the mean AUC exhibited a decline. Following the aforementioned RFA procedure, the final number of retained features was as follows: non-contrast phase: subregion 1 (11 features), subregion 2 (10 features), subregion 3 (14 features), entire tumor region (8 features); venous phase: subregion 1 (10 features), subregion 2 (13 features), subregion 3 (10 features), entire tumor region (16 features) (**Figure 3**).

### Model performance of clinical and subregional radiomics

The clinical data-based predictive model achieved AUCs of 0.778, 0.689, and 0.686 in the training, validation, and external test sets, respectively.

**Figure 4:**
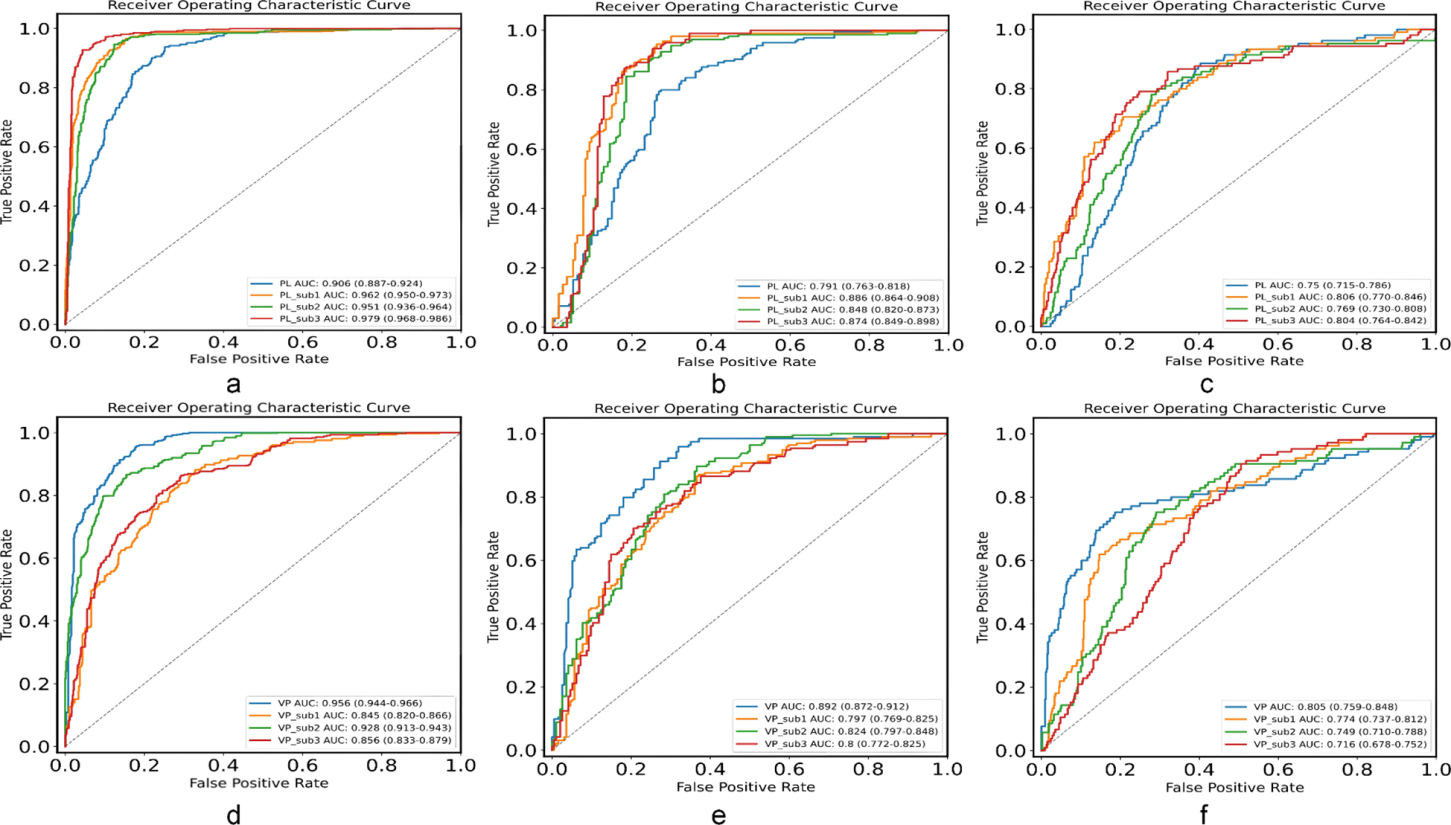
The ROC curves of the LR models in the **(a)** training, **(b)** validation, **(c)** external test set of unenhanced CT and **(d)** the training, **(e)** validation, **(f)** external test set of venous phase CT, respectively. ROC, receiver operating characteristic; LR, logistic regression.

Utilizing the radiomic features extracted from different subregions and the entire tumor region of the unenhanced and venous-phase CT, we constructed LR models. The model using features from the entire tumor region is described as the conventional radiomics model, and the ROC curves for each model are shown in **Figure 4**.

For both the training and testing datasets, the models based on the unenhanced CT phase subregions 1, 2, and 3 outperformed the conventional radiomics model. Wherein, the subregion 3 model achieved the highest AUC at 0.979 in the training set, with accuracy, sensitivity, and specificity of 97%, 98%, and 90%, respectively. It demonstrated the excellent performance in both validation and external testing sets, with AUC of 0.874 and 0.804 respectively.

Conversely, the performance of the conventional radiomics model derived from the CT venous phase was better than that of the subregions 1, 2, and 3 models in both the training and testing datasets. The conventional radiomics model recorded an AUC of 0.956 in the training set, with accuracy, sensitivity, and specificity of 94%, 97%, and 83%, respectively. It also indicated the robust performance in both validation and external testing sets, with AUC of 0.892 and 0.805, respectively.

**Figure 5:**
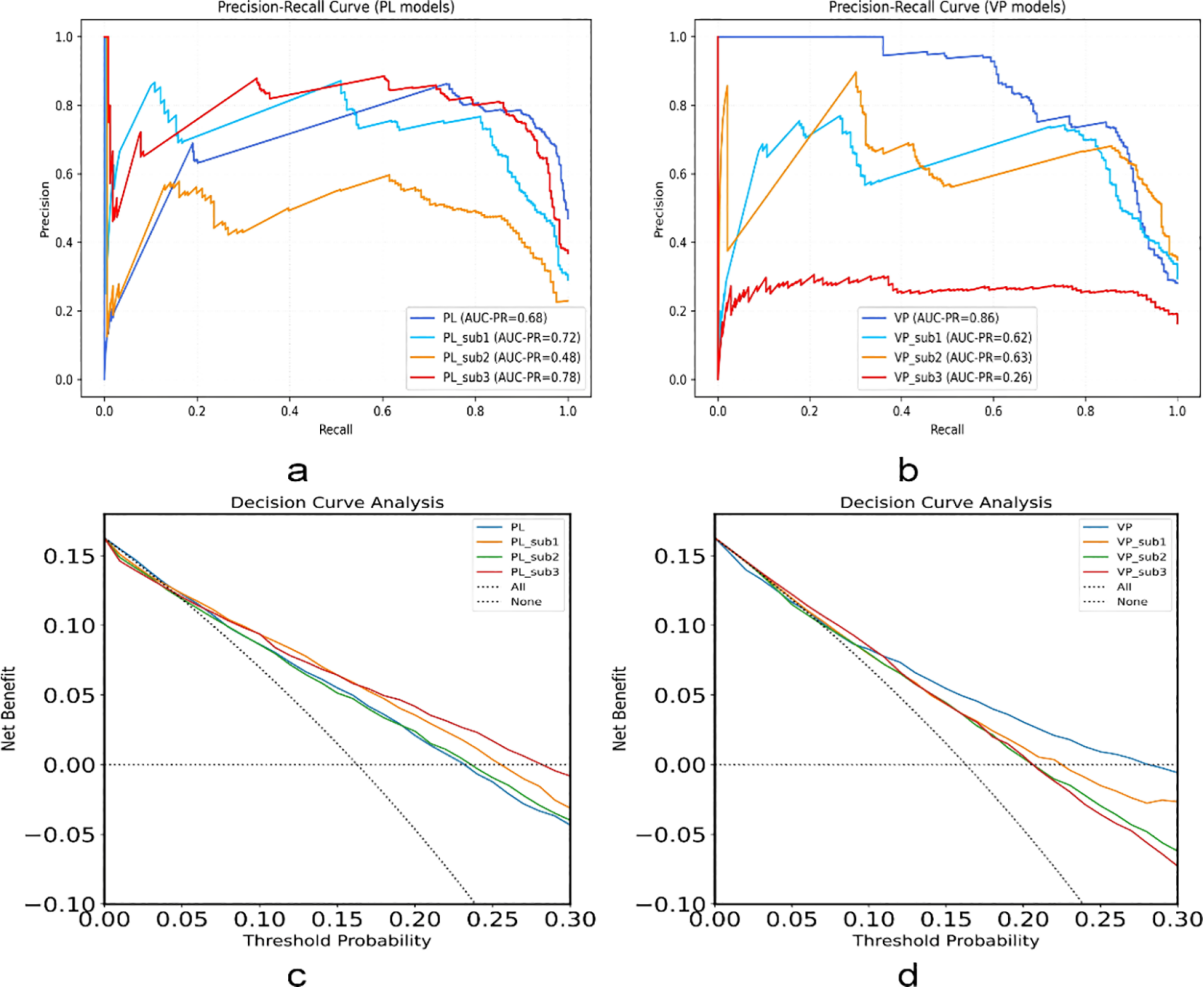
The precision-recall (P-R) analysis curves for different models in the external test set of **(a)** unenhanced CT and **(b)** venous phase CT, as well as the decision curve analysis (DCA) of the above models in **(c)** unenhanced CT and **(d)** venous phase CT. PL, plain scan/unenhanced scan; VP, venous phase; AUC-PR, area under the precision–recall curve.

The predictive performance of each model is presented in **Table 2**. The precision-recall (P-R) curves for various models using unenhanced and venous-phase CT in the external testing cohort, along with the DCA for these models, are shown in **Figure 5**. The results of correlation curve analysis demonstrated the significant net benefit from both the subregion 3 radiomics model in unenhanced CT phase and the conventional radiomics model in venous phase CT, emphasizing the clinical relevance of the model in predicting mitotic index in 2-5 cm gastric GISTs.

**Table 2:** The prediction performance of LR model in Center 1 and Center 2 dataset.

	Center 1												Center 2					
Item	Training						Validation						Test					
Model	AUC (95%CI)	ACC	SEN	SPE	TPR	FPR	AUC (95%CI)	ACC	SEN	SPE	TPR	FPR	AUC (95%CI)	ACC	SEN	SPE	TPR	FPR
Clinical	0.778 (0.683,0.872)	83%	17%	99%	17%	1.3%	0.689 (0.482,0.897)	83%	22%	97%	22%	2.6%	0.686 (0.551,0.820)	82%	9.5%	96%	9.5%	3.7%
Unenhanced scan																		
Subregion 1	0.962 (0.950,0.973)	94%	96%	87%	96%	13%	0.886 (0.864,0.908)	87%	93%	75%	93%	25%	0.806 (0.770,0.846)	67%	82%	64%	82%	36%
Subregion 2	0.951 (0.936,0.964)	95%	97%	87%	97%	13%	0.848 (0.820,0.873)	86%	93%	74%	93%	26%	0.769 (0.730,0.808)	67%	85%	64%	85%	36%
Subregion 3	0.979 (0.968,0.986)	97%	98%	90%	98%	10%	0.874 (0.849,0.898)	84%	88%	76%	88%	24%	0.804 (0.764,0.842)	75%	83%	73%	83%	27%
C-Radiomics	0.906 (0.887,0.924)	89%	91%	80%	91%	20%	0.791 (0.763,0.818)	78%	82%	70%	82%	24%	0.750 (0.715,0.786)	66%	88%	61%	88%	39%
Venous phase																		
Subregion 1	0.845 (0.820,0.866)	82%	84%	74%	84%	26%	0.797 (0.769,0.825)	76%	80%	69%	80%	31%	0.774 (0.737,0.812)	80%	68%	82%	68%	18%
Subregion 2	0.928 (0.913,0.943)	87%	88%	85%	88%	15%	0.824 (0.797,0.848)	78%	82%	71%	82%	29%	0.749 (0.710,0.788)	64%	84%	60%	84%	40%
Subregion 3	0.856 (0.833,0.879)	82%	84%	76%	84%	24%	0.800 (0.772,0.825)	74%	76%	71%	76%	29%	0.716 (0.678,0.752)	60%	79%	56%	79%	44%
C-Radiomics	0.956 (0.944,0.966)	94%	97%	83%	97%	17%	0.892 (0.872,0.912)	83%	88%	74%	88%	26%	0.805 (0.759,0.848)	79%	79%	78%	79%	22%

LR, logistic regression; C-Radiomics, conventional radiomics; AUC, area under the receiver operating characteristic curve; CI, confidence interval; ACC, accuracy; SEN, sensitivity; SPE, specificity. TPR, true positive rate; FPR, false positive rate.

### SHAP interpretability analysis

**Figure 6:**
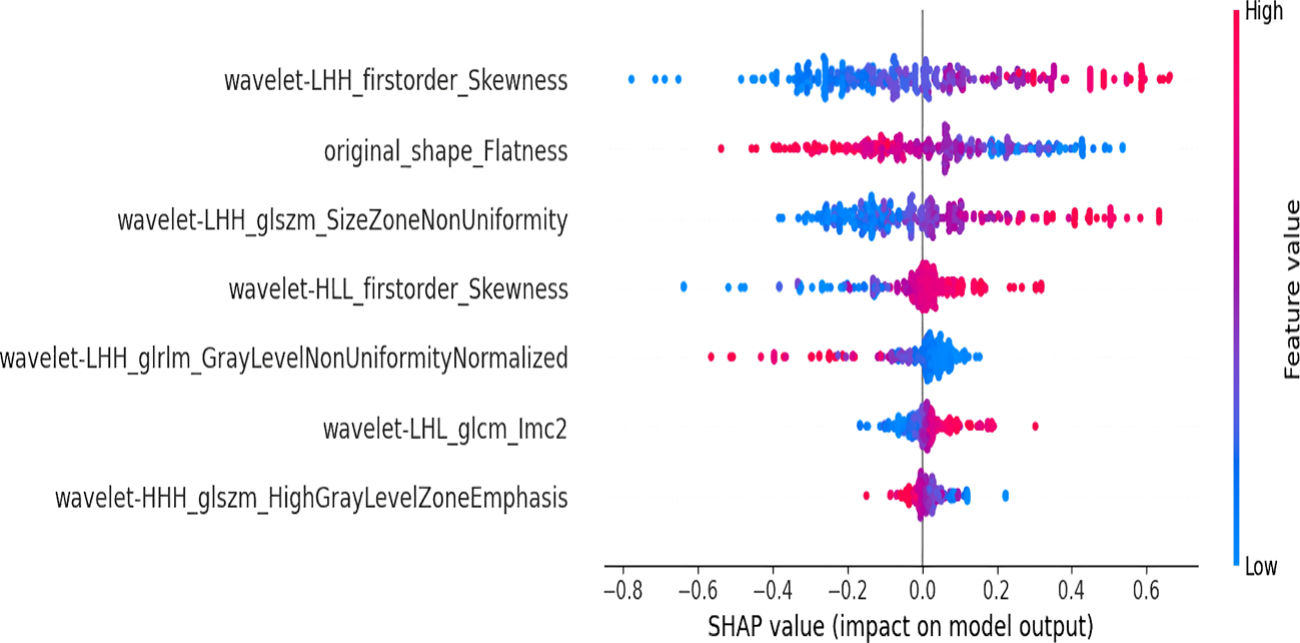
SHAP plots of the top 7 radiomic features from the CT non-contrast subregion 3 model. Features located higher have a greater impact on the model’s prediction. Red indicates higher feature values, while blue indicates lower ones. SHAP values below 0 are associated with the low mitosis group in this study, whereas values above 0 are linked to the high mitosis group. In other words, higher SHAP values increase the likelihood of predicting the high mitosis group.

Among all radiomics models, the subregion 3 model based on unenhanced CT demonstrate excellent predictive value. Consequently, SHAP interpretability analysis was performed on this model (**Figure 6**), providing a comprehensive explanation of the radiomic features. The unenhanced CT subregion 3 model was largely driven by two key features: wavelet-LHH_firstorder_Skewness, reflecting the asymmetry of voxel intensity distribution, and original_shape_Flatness, characterizing the lesion’s spatial geometry. Greater prominence of these features biases the model toward the high-mitosis group.

## Discussion

**Figure 7:**
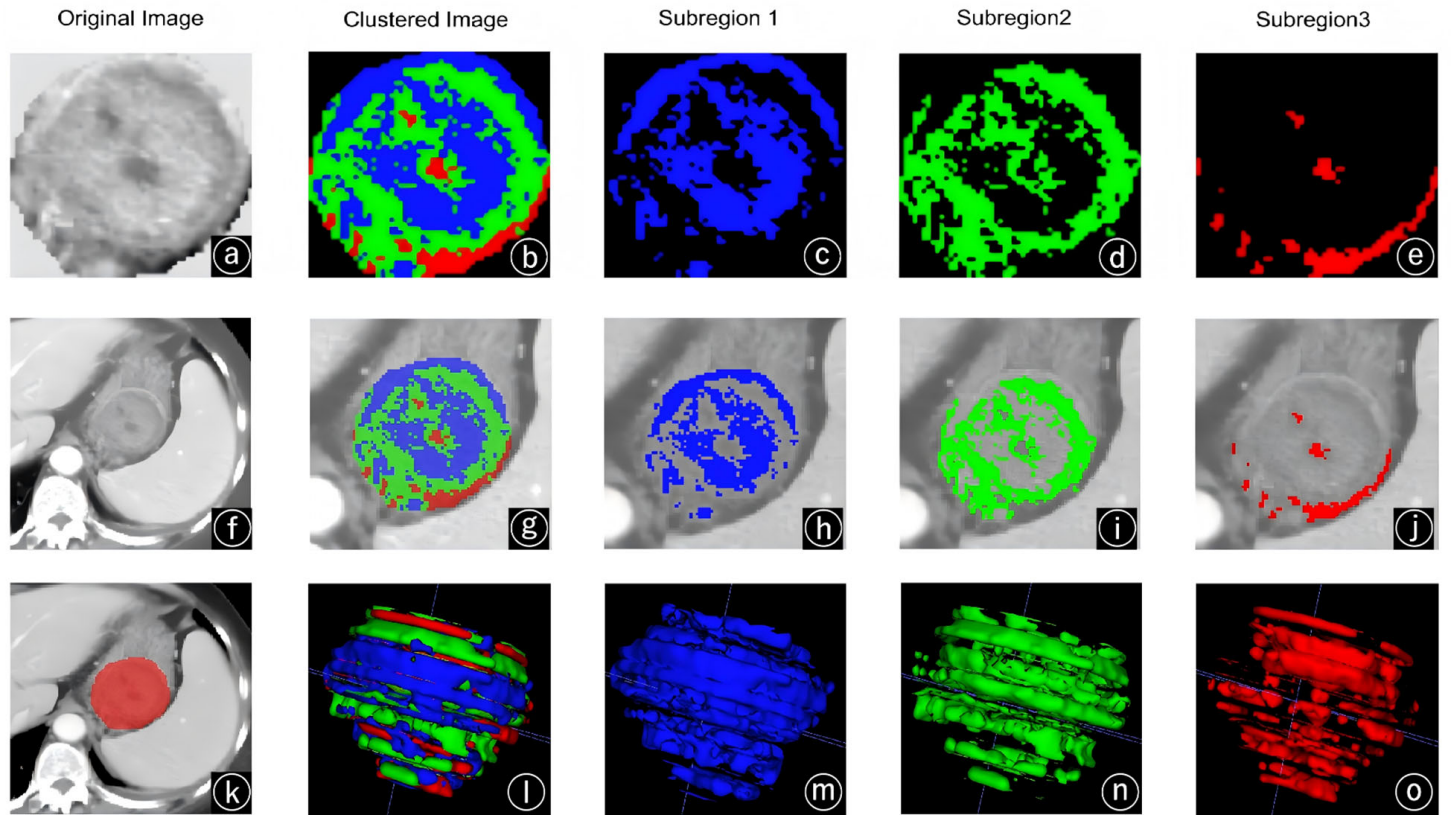
Demonstration of the clustering process. The tumor ROI image features **(a)** are clustered to obtain clustered images **(b)**. The clustered images are divided into tumor subregion 1 **(c)**, subregion 2 **(d)**, and subregion 3 **(e)**. Mapping these subregions onto CT image **(g–j)** indicates that subregion 1 **(h)** is predominantly located in high-density lesion areas, while subregion 3 **(j)** is mainly found in low-density regions. Figure **(f)** is the original CT image, figure **(k)** is the ROI delineation diagram, and figures (l, m n, o) are the 3D of ROI.

This study is the first attempt to apply a subregional radiomics method to explore the mitotic index and risk stratification in patients with 2-5 cm gastric GISTs. We ultimately formed three subregions through clustering and mapped the segmented image onto the original CT images. The results indicated that subregion 1 was primarily concentrated in areas with higher lesion density, whereas subregion 3 was mainly located in areas with lower density, respectively (**Figure 7**). Previous studies have demonstrated that the density of non-contrast-enhanced CT is correlated with the intratumoral tissue components. The hyperdense regions on contrast-enhanced CT correspond to areas with high tumor cell viability and dense vascular distribution. Mapping results indicate that our clustering outcomes are associated with intratumoral heterogeneity (28).

In the unenhanced CT-based radiomics models, the performance of models constructed using subregion 1, subregion 2, and subregion 3 outperformed both conventional radiomics models and clinical models in both the training set and external test set. Specifically, the AUC of the subregion 3 model reached 0.979 in the training set, and it maintained robust performance in the validation set and external test set (AUC = 0.874 and 0.804, respectively). These findings demonstrate that unenhanced CT images of GISTs patients have clinical utility for mitotic count prediction—particularly in those with contraindications to iodine-based contrast agents, such as iodine allergy, hyperthyroidism, severe liver or kidney impairment, significant heart failure, or renal failure. The subregional radiomics approach, which is based on unenhanced CT, provides a novel perspective for tumor risk assessment and therapeutic efficacy prediction.

Cai et al. (21) utilized enhanced CT-based tumor subregional radiomics to predict the Ki-67 proliferation index in GISTs, revealing the good performance of the subregional prediction model. They found that the subregion 1 model outperformed the conventional radiomics model. However, in this study, the whole-tumor model in the venous phase demonstrated superior predictive performance compared with the subregional models, while the latter still exhibited robust predictive capability. We speculated that this discrepancy might stem from our focus specifically on the mitotic index and risk stratification of 2-5 cm gastric GISTs and the exclusion of cases of non-gastric tumors and gastric GISTs larger than 5 cm. Furthermore, 2-5 cm gastric GISTs typically exhibit higher relative size and density consistency and are generally at a lower risk for necrosis, cystic degeneration, or rupture and hemorrhage compared to larger tumors. The blood supply to these smaller tumors tends to be more homogeneous, leading to lower internal heterogeneity. Additionally, the introduction of contrast agents may mask intratumoral heterogeneity, thereby potentially limiting the performance improvement of the subregional radiomics model.

In this study, we found that the AUC values of subregional CT radiomics from unenhanced scans in the external testing cohort were slightly higher than those from venous-phase subregional CT radiomics. Zhang et al. (17) reported that the non-contrast radiomics model was more effective in assessing the malignant risk of GISTs, with AUCs of 0.967 and 0.960 in internal and external validation, respectively, outperforming those of the contrast-enhanced model (AUCs: 0.941 and 0.899). Our findings are consistent with this result. We hypothesize that this phenomenon arises from individual differences in constitution, metabolism, and tumor perfusion, which lead to heterogeneity in the pharmacokinetics of the contrast agent, thereby altering subregional imaging textures and generating radiomic features that differ from those on non-contrast images. Gastric GISTs in the 2–5 cm range are often more homogeneous than larger tumors, and their uniform contrast enhancement may obscure or homogenize subtle internal heterogeneity. Conversely, non-contrast images may reveal the tumor’s intrinsic density distribution and internal texture, which may be more directly associated with its cellular composition and mitotic activity. Although the conclusions drawn from this study are reasonable to certain extent, they remain speculative, and the underlying mechanisms warrant further investigation.

In our study, SHAP analysis revealed several radiomic features that were critical for predicting the malignant potential of GISTs. Among them, Wavelet-LHH-first order-Skewness and Flatness contributed strongly. Skewness measures the degree of asymmetry in the distribution of pixel values in an image, which may be associated with intratumoral heterogeneity. A higher ratio of intratumoral necrosis or cystic change is associated with a greater likelihood of a high mitotic, potentially resulting in more pronounced skewness values (29). Flatness quantifies how much the tumor morphology deviates from a perfect sphere. Higher flatness values indicate a more flattened and irregular tumor morphology, which may be associated with the infiltrative growth pattern of GISTs with high malignant potential (30).

Yang et al. (12) conducted an analysis of imaging features in a single-center cohort of 233 GIST patients. Their results demonstrated that these features had good predictive value for risk stratification of 2-5 cm gastric GISTs, with an AUC of 0.82. Furthermore, in another study aimed at predicting the malignant potential of 2-5 cm gastric GISTs using CT radiomics, a model developed from the arterial-phase images of 103 patients from the same institution demonstrated high predictive performance, with AUCs of 0.919 in the training set versus 0.881 in the test set (31). However, these previous studies were limited by small sample sizes and a lack of external validation. Our study filled these gaps, and the model we developed demonstrated satisfactory predictive ability and generalizability.

Our study has some limitations. First, being a retrospective study, a certain degree of selection bias is present. Second, although our study enrolled a larger cohort than previous studies, the proportion of samples with high mitotic counts may still be insufficient. Furthermore, 2-5 cm gastric GISTs are often relatively homogeneous lesions, which may limit the biological significance of subregional analysis. These factors could potentially affect the model’s predictive performance. Therefore, future studies should employ larger-scale, prospective, multi-center validation to ensure generalizability. However, the performance of the model in the validation set closely mirrored that in the external testing set, indicating its robustness. Third, this study only extracted features from unenhanced CT and venous-phase scans. In the future, we plan to incorporate arterial- and delayed-phase images to obtain a more comprehensive characterization of tumor heterogeneity and acquire richer radiomic features for model construction. Fourth, we did not correlate CT qualitative and quantitative parameters with radiomics. Future studies should consider additional cohort validations and more factors that can be integrated for further exploration. Fifth, this study utilized a manual ROI segmentation method, and small segmentation errors in image processing might have affected the presence or absence of certain subregions.

In conclusion, the results of this study indicate that the subregional CT radiomics model is an innovative and reliable tool for the preoperative prediction of mitotic levels in patients with 2-5 cm gastric GISTs. Significantly, the subregional model based on unenhanced CT demonstrated strong predictive performance. Subregional radiomics can serve as a potential non-invasive clinical assessment tool, providing new perspectives and additional information for risk stratification of GISTs, and potentially contributing to personalized clinical decision-making for patients in the future.

## Data Availability

The original contributions presented in the study are included in the article/supplementary material. Further inquiries can be directed to the corresponding authors.
